# The impact of sport-specific physical fitness change patterns on lower limb non-contact injury risk in youth female basketball players: a pilot study based on field testing and machine learning

**DOI:** 10.3389/fphys.2023.1182755

**Published:** 2023-05-12

**Authors:** Yuanqi Huang, Changfei Li, Zhanshuang Bai, Yukun Wang, Xiaohong Ye, Yuheng Gui, Qiang Lu

**Affiliations:** ^1^ School of Science, Jimei University, Xiamen, China; ^2^ School of Physical Education and Sport Science, Fujian Normal University, Fuzhou, China; ^3^ Fujian Provincial Basketball and Volleyball Centre, Fuzhou, China; ^4^ School of Tourism and Sports Health, Hezhou University, Hezhou, China; ^5^ Institute of Sport Business, Loughborough University London, London, United Kingdom; ^6^ Chengyi College, Jimei University, Xiamen, China

**Keywords:** injury management, physical fitness, data science, injury risk pattern, machine learning

## Abstract

**Background:** In recent years, identifying players with injury risk through physical fitness assessment has become a hot topic in sports science research. Although practitioners have conducted many studies on the relationship between physical fitness and the likelihood of injury, the relationship between the two remains indeterminate. Consequently, this study utilized machine learning to preliminary investigate the relationship between individual physical fitness tests and injury risk, aiming to identify whether patterns of physical fitness change have an impact on injury risk.

**Methods:** This study conducted a retrospective analysis by extracting the records of 17 young female basketball players from the sport-specific physical fitness monitoring and injury registration database in Fujian Province. Sports-specific physical fitness tests included physical performance, physiological, biochemical, and subjective perceived responses. The data for each player was standardized individually using Z-scores. Synthetic minority over-sampling techniques and edited nearest neighbor algorithms were used to sample the training set to address the negative impact of class imbalance on model performance. Feature extraction was performed on the dataset using linear discriminant analysis, and the prediction model was constructed using the cost-sensitive neural network.

**Results:** The 10 replicate 5-fold stratified cross-validation showed that the lower limb non-contact injury prediction model based on the cost-sensitive neural network had achieved good discrimination and calibration (average Precision: 0.6360; average Recall: 0.8700; average F2-Score: 0.7980; average AUC: 0.8590; average Brier-score: 0.1020), which could be well applied in training practice. According to the attribution analysis, agility and speed were important physical attributes that affect youth female basketball players’ non-contact lower limb injury risk. Specifically, there was enhance in the performance of the 1-min double under, accompanied by an increase in urinary ketone and urinary blood levels following the agility test. The 3/4 basketball court sprint performance improved, while urinary protein and RPE levels decreased after the speed test.

**Conclusion:** The sport-specific physical fitness change pattern can impact the lower limb non-contact injury risk of young female basketball players in Fujian Province, specifically in terms of agility and speed. These findings will provide valuable insights for planning athletes’ physical training programs, managing fatigue, and preventing injuries.

## 1 Introduction

Sports injuries are a common condition in basketball that considerably impact sports performance, health, and team finances. Previous studies have shown that the incidence of sports injuries is increasing year after year. The investigation by Meeuwisse et al. (2003) reported that training injuries accounted for 70% of sports injuries in basketball players, with 47% of training injuries not due to physical contact. Of these non-contact injuries, approximately 28% would result in the player being unable to participate in training for a short time. In a recent study, the average weekly injury rate among youth female basketball players was 20.7%, with approximately 58% of these injuries being non-contact (Sommerfield et al., 2020). It can be seen that most of the injuries are not due to physical contact, which means that they can be prevented, at least to a certain extent. Therefore, limiting the incidence of injury is a significant concern of the sports industry, sports science, and sports medicine, which is essential to maximize the effectiveness of sports training (Drew et al., 2017; Kester et al., 2017).

Basketball is a high-intensity intermittent team sport that combines speed, agility, strength, and speed endurance, which requires players to have a high level of physical fitness and sport-specific skills (Fort-Vanmeerhaeghe et al., 2016). If a player lacks high levels of speed, strength, agility, and speed endurance, it will be challenging to perform competitively in intense on-court match-ups, increasing the likelihood of injury risk (Hrysomallis, 2007; Herman et al., 2012; Lauersen et al., 2013; Emery et al., 2015). When injuries occur to these fewer fitness players, it takes them longer to recover their fitness levels (McGuigan, 2017). Therefore, identifying the potential risk of injury according to players’ physical fitness and intervening in time is an important practical issue. Although numerous studies have reported the association between physical fitness and injury risk in Australian football, rugby, and football (Harrison and Johnston, 2017; Macmillan et al., 2021; Leppänen et al., 2022), the findings are contradictory due to factors such as age, gender, sport-specific, and testing protocol. For example, cross-sectional studies have shown that better speed and strength characteristics were associated with a lower risk of injury (Gabbett et al., 2012; Gastin et al., 2015; Watson et al., 2017). Meanwhile, Some scholars showed no association between aerobic capacity, strength, speed, agility, and injury risk (Arnason et al., 2004; Emery et al., 2005; Kennedy et al., 2012). Furthermore, the studies by Quarrie et al. (2001) and Henderson et al. (2010) showed that higher levels of speed and strength performance were associated with higher injury risk. These conflicting findings make the regularity between physical fitness and injury risk unclear, particularly in basketball, where is limited research on youth players (Chang and Lu, 2020).

Through comparative analysis of current research, three limitations of this research area can be noted. Firstly, assessing a player’s physical fitness solely based on physical performance is a limited approach. Some research evidence showed that the physical fitness level strongly correlates with the player’s physiological status. The investigation by McLean et al. (2010) found significant differences in physiological responses between players with similar physical performance. Meanwhile, Clemente-Suárez et al. (2021) research reported differences in physical performance between players with different physiological statuses. So far, only a few studies have been reported to investigate the relationship between physical fitness and injury risk by combining physical performance with physiological indicators (e.g., maximal oxygen uptake). Unfortunately, these studies have not combined physical performance with post-test physiological, biochemical (e.g., blood lactate and creatine kinase), and subjective perceived responses for co-analysis (Eliakim et al., 2018). Secondly, most studies used cross-sectional study designs to investigate the relationship between physical performance and injury risk, which may be logically flawed. According to Simpson’s paradox, the correlations obtained this way are only relationships at the overall level and do not reflect correlations for each individual (Mangalam and Kelty-Stephen, 2021). Therefore, the correlation obtained through cross-sectional research is challenging to apply in training guidance. To address this issue, some scholars have conducted prospective cohort studies on the relationship between physical fitness and injury risk. Bennett et al. (2022) explored the relationship between physical fitness and injury risk by collecting four consecutive seasons of physical performance indicators and weekly injury status for Australian football players to obtain more accurate results. However, it is essential to note that the study’s physical performance indicators were collected before the season, meaning that changes in players’ physical fitness through different training stages were not fully considered, which may make it challenging to apply the findings to staged training programs. Thirdly, most studies used univariate statistical association strategies for analysis, which may have methodological shortcomings. Ruddy et al. (2019) pointed out that univariate statistical association methods can clarify the direct effects between measure variables and injury risk, which only obtain information on certain factors in the injury risk pattern and do not identify the injury risk pattern as a whole. In addition, this statistical strategy is challenging to identify the non-linear relationship between independent and dependent variables and the interaction between variables. Moreover, the extreme value of samples also dramatically influences the model, so there are still some methodological limitations.

Consequently, based on the above-mentioned limitations, this study employs the machine learning approach to preliminary investigate the relationship between sport-specific physical fitness and injury risk. The objective is to determine whether changes in an individual’s sport-specific physical fitness impact their injury risk and to identify patterns in physical fitness changes associated with injury risk. This study collected data on sport-specific physical fitness monitoring and injury registration of youth female basketball players in Fujian Province. The machine learning algorithm was used to construct a lower limb non-contact injury risk prediction model and to discover their injury risk pattern. The findings will help coaches and researchers to understand players’ lower limb non-contact injury risk and to predict lower limb non-contact injury risk through the data-driven model.

## 2 Materials and methods

The data used in this study were obtained from the sport-specific physical fitness monitoring and injury registration database of youth female basketball players established by the Fujian Provincial Basketball and Volleyball Sports Management Centre during the preparation for the 13th National Games. The database recorded data for seventeen youth female basketball players (age: 15.00 ± 0.61 years; height: 176.58 ± 6.46 cm; weight: 64.42 ± 8.47 kg; training year: 1.62 ± 0.65 years) between January and December 2014. All players were affiliated with Fujian Provincial Basketball and Volleyball Sports Management Centre. Prior to participation, all players provided informed consent, and the study was conducted in accordance with the Declaration of Helsinki guidelines. The Fujian Province Basketball and Volleyball Sports Management Centre approved the study. These data were collected by the Fujian Provincial Basketball and Volleyball Sports Management Centre and shared with the researchers involved in this study through a non-disclosure agreement. The center has the right to choose which information, results, and data can be made publicly available and grant access to these data for research purposes only to the authors of this paper.

### 2.1 Data collection

The dataset used in this study evaluated sport-specific physical fitness from four dimensions: physical performance, physiological, biochemical, and subjective perceived response.

#### 2.1.1 Physical performance

The database’s sport-specific physical performance test protocol was based on the Chinese Youth Basketball Training Syllabus. The 15 m × 13 shuttle run test was selected to assess the speed endurance of the players. The 3/4 basketball court sprint test assessed the player’s speed. The 1-minute double under and hexagon agility test assessed the player’s agility. The 30-second 35 kg squat, 30-second 20 kg bench press, 30-second sit-up, and 30-second back extensions test assessed the player’s strength. Data were collected at 6-week intervals for a total of 6 sessions. Further information can be found in Supplementary File S1.

#### 2.1.2 Physiological response

The dataset used in this study contains players’ physiological responses after physical performance tests. According to Fujian Provincial Basketball and Volleyball Sports Management Centre test records, players will be asked to test the instantaneous heart rate and heart rate recovery (heart rate at 1 min after testing) after the speed, speed endurance, and agility test. Instantaneous heart rate (IHR) and heart rate recovery (HRR) were acquired using the Polar team telemetry heart rate device.

#### 2.1.3 Biochemical response

The dataset contains the biochemical responses of the players after the physical fitness test. Players were required to measure creatine kinase immediately after the strength test and the following day, and blood lactate at 3 min after the speed endurance test. Within 15 min of each physical performance test completion, the player’s first mid-phase urinary was tested for urinary composition using the CLINITEK STATUS Urinary Decathlon Analyser. The urinary protein, urinary specific gravity, urinary blood, urobilinogen, pH, and urinary ketones were assigned to the urinary components (Table 1). The Medical Department of Fujian Provincial Basketball and Volleyball Sports Management Centre did all measurements.

**TABLE 1:** Assignment rules and independent variable encoding of physical performance, physiological, biochemical, and subjective perceived response indicators for each physical fitness attribute.

Physical fitness attributes	Indicator	Assignment	Encoding
Agility	1-minute double under	Original value input	A1
	1-minute double under IHR (1min)	Original value input	A2
	1-minute double under HRR (1min)	Original value input	A3
	hexagon agility test	Original value input	A4
	hexagon agility test IHR (1min)	Original value input	A5
	hexagon agility test HRR (1min)	Original value input	A6
	RPE	Original value input	A7
	urinary protein	Negative = 1; Microscale = 2; 0.3 g/L = 3; 1 g/L = 4; 3 g/L = 5	A8
	urobilinogen	3.2 mg/dL = 1; 16 mg/dL = 5; 33 mg/dL = 10	A9
	urinary-PH	Original value input	A10
	urinary specific gravity	≤1.025 = 1; ≥1.030 = 2	A11
	urinary blood	Negative = 1; Microscale = 2; Ca25 Ery/µL = 3; Ca80 Ery/µL = 4; Ca200 Ery/µL = 5	A12
	urinary ketones	Negative = 1; Microscale = 2; 1.5 nmol/L = 3	A13
Speed Endurance	15 m × 13 shuttle run time	Original value input	B1
	15 m × 13 shuttle run IHR	Original value input	B2
	15 m × 13 shuttle run HRR (1min)	Original value input	B3
	15 m × 13 shuttle run BLA (3min)	Original value input	B4
	RPE	Original value input	B5
	urinary protein	Negative = 1; Microscale = 2; 0.3 g/L = 3; 1 g/L = 4; 3 g/L = 5	B6
	urobilinogen	3.2 mg/dL = 1; 16 mg/dL = 5; 33 mg/dL = 10	B7
	urinary-PH	Original value input	B8
	urinary specific gravity	≤1.025 = 1; ≥1.030 = 2	B9
	urinary blood	Negative = 1; Microscale = 2; Ca25 Ery/µL = 3; Ca80 Ery/µL = 4; Ca200 Ery/µL = 5	B10
	urinary ketones	Negative = 1; Microscale = 2; 1.5 nmol/L = 3	B11
Strength	30-second 35 kg squat	Original value input	C1
	30-second 20 kg bench press	Original value input	C2
	30-second sit up	Original value input	C3
	30-second back extensions	Original value input	C4
	CK change	Original value input	C5
	RPE	Original value input	C6
	urinary protein	Negative = 1; Microscale = 2; 0.3 g/L = 3; 1 g/L = 4; 3 g/L = 5	C7
	urobilinogen	3.2 mg/dL = 1; 16 mg/dL = 5; 33 mg/dL = 10	C8
	urinary-PH	Original value input	C9
	urinary specific gravity	≤1.025 = 1; ≥1.030 = 2	C10
	urinary blood	Negative = 1; Microscale = 2; Ca25 Ery/µL = 3; Ca80 Ery/µL = 4; Ca200 Ery/µL = 5	C11
	urinary ketones	Negative = 1; Microscale = 2; 1.5 nmol/L = 3	C12
Speed	3/4 basketball court sprint time	Original value input	D1
	RPE	Original value input	D2
	urinary protein	Negative = 1; Microscale = 2; 0.3 g/L = 3; 1 g/L = 4; 3 g/L = 5	D3
	urobilinogen	3.2 mg/dL = 1; 16 mg/dL = 5; 33 mg/dL = 10	D4
	urinary-PH	Original value input	D5
	urinary specific gravity	≤1.025 = 1; ≥1.030 = 2	D6
	urinary blood	Negative = 1; Microscale = 2; Ca25 Ery/µL = 3; Ca80 Ery/µL = 4; Ca200 Ery/µL = 5	D7
	urinary ketones	Negative = 1; Microscale = 2; 1.5 nmol/L = 3	D8

Abbreviations: IHR, instantaneous heart rate; HRR, heart rate recovery; RPE, ratings of perceived exertion; BLA, blood lactate; CK: creatine kinase.

#### 2.1.4 Subjective perceived response

This study used the Borg-10 ratings of perceived exertion (RPE) scale designed by Foster et al. (1995) to quantify the perceived exertion level of players after a physical performance test. Numerous studies have confirmed this quantification method’s validity and reliability Chen et al. (2002). Within 10 min of completing the test, the researcher verbally asks the players about their fatigue level.

#### 2.1.5 Injury registration

Based on the injury data collection procedure (Fuller et al., 2006), medical staff from the Fujian Province Basketball and Volleyball Sports Management Centre diagnosed injuries through medical examination, medical imaging diagnosis, and other methods. The injury registry recorded location, type, injury occurrence (contact, non-contact), and diagnosis mode. Following the definition of Bahr et al. (2020), Non-contact injuries of lower limbs were defined as those caused by mechanisms other than direct contact, including overuse injuries and chronic injuries. Following the definition of Enright et al. (2019), the lower limb includes the hips, thighs, knees, calves, ankles, and feet.

### 2.2 Data processing

The missing values in the dataset were analyzed using the SPSS 26.0 software, multiple imputations were performed at the individual level for independent variables with no more than 10% missing values, and independent variables with more than 10% missing values were excluded. According to research reports (Hubal et al., 2005; Gabbett and Domrow, 2007; Buford et al., 2013; Halson, 2014), there are significant differences in the development potential of physical fitness among players, so this study employs the within-individual difference approach to perform the numerical transformation of panel data. The independent variables for each player were standardized using the Z-score transformation. Numerous studies have reported that the occurrence of sports injuries in the real world tends to show a skewed distribution (Rossi, 2017; Rossi et al., 2018), which may lead to machine learning models not correctly classifying the minority class samples (injury samples). In addition, some samples may harm the model performance, which will make the classification boundary between the majority and minority samples may be blurred. Therefore, the synthetic minority over-sampling techniques and edited nearest neighbor (SMOTEENN) algorithm were used to sample training sets in each folding of cross-validation to reduce the impact of class imbalance on model training (Manju and Nair, 2019).

### 2.3 Feature extraction

Multi-dimensional evaluation can provide players’ physical fitness information to practitioners more comprehensively. However, more independent variables mean more complex models, making the model more prone to over-fitting under limited data. Consequently, this study employed the linear discriminant analysis (LDA) algorithm to diminish the dimensional of the training set based on four distinct dimensions: strength, speed, agility, and speed endurance.

### 2.4 Model construction

This study used a cost-sensitive neural network algorithm (Cost-NN) to construct a prediction model for lower limb non-contact injury risk. The network model consists of an input layer, a hidden layer, a dropout layer, and an output layer. The adaptive moment estimation (Adam) was used in this study to optimize the network by minimizing the binary cross entropy of the independent and dependent variables. Meanwhile, the validation set was also used for parameter adjustment, and training was stopped early when the precision of the validation set decreased for more than 30 iterations. This study set the training parameter epoch to 100. To illustrate that the Cost-NN used in this study can effectively identify patterns of non-contact injury risk in the lower limbs of youth female basketball players. This study constructed the dummy classifier (DC) as a model performance baseline, randomly assigning a category to a sample while respecting the category distribution. The model was also compared to logistic regression (LR), random forest (RF), extreme gradient boosting (XGBoost), balanced random forest (BRF), and random undersampling Adaboost (RUSBoost) algorithms used in research reports (López-Valenciano et al., 2017; Ruddy et al., 2018; Bryan et al., 2020; Jauhiainen et al., 2020; Rommers et al., 2020). The model was built and evaluated using a 5-fold stratified cross-validation with 10 repeated iterations. Metrics such as precision, recall, the area under the receiver operating characteristic curve (AUC), and the F2-score were used to evaluate the model’s discrimination. The Brier score was used to evaluate the probabilistic calibration of the model. The decision curve analysis was performed for the clinical applicability of the model.

### 2.5 Model interpretation

Since the model in this study was constructed through cross-validation, which means that multiple models were generated, the Wald χ^2^ test was used to estimate the significance of the discriminant coefficients. The Wald χ^2^ test was conducted using a two-sided hypothesis test with the significance level set (*α*) at 0.05 and considered *p* > 0.1 as insignificant, *p* < 0.1 as marginally significant, and *p* < 0.05 as significant. In addition, the independent variables in the LDA and the Cost-NN were analyzed using the Shapley additive explanations (SHAP) method, and the relative feature importance of each independent variable was calculated (Nohara et al., 2019). The injury risk pattern analysis was performed by calculating the mean relative importance of the independent variables in LDA and Cost-NN. The above model construction, validation, and important analysis were performed in the Python 3.6 programming environment.

## 3 Results

Through the missing value analysis, this study included 84 valid data samples, of which 18 data samples occurred with lower limb non-contact injuries in the next 6 weeks. The mean values and 95% confidence intervals for each player were described in Supplementary File S2 **.**

Since each physical fitness attribute was assessed comprehensively from four aspects: physical performance, physiological, biochemical, and subjective perceived response, LDA was used for dimensional reduction separately for each physical fitness attribute. The feature variables of each physical fitness attribute were obtained by extracting the discriminant coefficients of the LDA function of each fold in the cross-validation and calculating the average value of the discriminant coefficients. The discriminant formula of variables is shown in Table 2.

**TABLE 2:** The discriminant coefficient and significance of the LDA function for each physical attribute.

Physical fitness attributes	Indicator	Encoding	Coef	SE	Wald	*p*
Agility	1-minute double under	A1	1.792	0.720	6.195	0.013**
	1-minute double under IHR (1min)	A2	−0.853	0.656	1.691	0.194
	1-minute double under HRR (1min)	A3	−1.921	0.470	16.705	<0.001***
	hexagon agility test	A4	0.948	0.433	4.793	0.029**
	hexagon agility test IHR (1min)	A5	2.563	0.525	23.833	<0.001***
	hexagon agility test HRR (1min)	A6	1.902	0.704	7.299	0.007***
	RPE	A7	−1.807	0.399	20.510	<0.001***
	urinary protein	A8	2.439	0.559	19.037	<0.001***
	urobilinogen	A9	2.319	0.443	27.403	<0.001***
	urinary-PH	A10	0.654	0.414	2.495	0.114
	urinary specific gravity	A11	1.156	0.413	7.835	0.005***
	urinary blood	A12	5.306	0.728	53.122	<0.001***
	urinary ketones	A13	5.698	0.659	74.761	<0.001***
Speed Endurance	15 m × 13 shuttle run time	B1	1.230	0.294	17.503	<0.001***
	15 m × 13 shuttle run IHR	B2	−0.506	0.304	2.770	0.096*
	15 m × 13 shuttle run HRR (1min)	B3	1.505	0.271	30.841	<0.001***
	15 m × 13 shuttle run BLA (3min)	B4	−1.407	0.340	17.125	<0.001***
	RPE	B5	−3.469	0.274	160.290	<0.001***
	urinary protein	B6	1.582	0.443	12.753	<0.001***
	urobilinogen	B7	0.010	0.384	0.001	0.978
	urinary-PH	B8	−0.964	0.394	5.986	0.014**
	urinary specific gravity	B9	−1.046	0.298	12.321	<0.001***
	urinary blood	B10	0.842	0.281	8.979	0.003***
	urinary ketones	B11	0.524	0.311	2.839	0.092*
Strength	30-second 35 kg squat	C1	−1.440	0.260	30.675	<0.001***
	30-second 20 kg bench press	C2	−1.529	0.278	30.250	<0.001***
	30-second sit up	C3	0.692	0.233	8.821	0.003***
	30-second back extensions	C4	0.393	0.211	3.469	0.062**
	CK change	C5	3.681	0.288	163.360	<0.001***
	RPE	C6	−2.565	0.351	53.402	<0.001***
	urinary protein	C7	−2.521	0.272	85.903	<0.001***
	urobilinogen	C8	1.716	0.330	27.040	<0.001***
	urinary-PH	C9	−1.959	0.273	51.492	<0.001***
	urinary specific gravity	C10	−1.357	0.308	19.411	<0.001***
	urinary blood	C11	1.459	0.206	50.162	<0.001***
	urinary ketones	C12	−0.301	0.272	1.225	0.270
Speed	3/4 basketball court sprint time	D1	−3.940	0.257	235.032	<0.001***
	RPE	D2	−2.119	0.204	107.895	<0.001***
	urinary protein	D3	−2.459	0.229	115.304	<0.001***
	urobilinogen	D4	0.461	0.261	3.120	0.077*
	urinary-PH	D5	−0.654	0.129	25.703	<0.001***
	urinary specific gravity	D6	−0.800	0.160	25.000	<0.001***
	urinary blood	D7	2.537	0.260	95.213	<0.001***
	urinary ketones	D8	−2.292	0.205	125.003	<0.001***

*p < 0.1, **p < 0.05, ***p < 0.01.

Abbreviations: IHR, instantaneous heart rate; HRR, heart rate recovery; RPE, ratings of perceived exertion; BLA, blood lactate; CK, creatine kinase.

The mean and 95% confidence intervals of the precision, recall, F2-score, AUC, and Brier score of the model were evaluated in this study by 5-fold cross-validation with 10 replicates (Table 3). As shown in Table 3, compared with the baseline and commonly used models in research reports, the lower limb non-contact injury risk prediction model constructed based on Cost-NN has better discrimination and probabilistic calibration, which indicates that the model was effective in identifying potential risk patterns for lower limb non-contact injury in the next 6 weeks.

**TABLE 3:** Evaluation results of the model’s discrimination and calibration performance metrics.

Model	Precision		Recall		F2-score		AUC		Brier-score	
	Mean	95% CI	Mean	95% CI	Mean	95% CI	Mean	95% CI	Mean	95% CI
DC	0.1930	0.1365–0.2500	0.1880	0.1303–0.2460	0.1800	0.1258–0.2340	0.4840	0.4536–0.5130	0.3110	0.2824–0.3390
LR	0.4280	0.3905–0.4650	0.7720	0.7175–0.8260	0.6530	0.6108–0.6960	0.7350	0.7023–0.7670	0.2090	0.1913–0.2270
RF	0.5270	0.4376–0.6160	0.4230	0.3470–0.5000	0.4270	0.3542–0.4990	0.6630	0.6239–0.7010	0.1340	0.1224–0.1460
BRF	0.4560	0.4076–0.5040	0.8000	0.7468–0.8530	0.6780	0.6337–0.7230	0.7530	0.7204–0.7860	0.1800	0.1634–0.1960
XGBoost	0.4370	0.3921–0.4810	0.7170	0.6512–0.7820	0.6220	0.5698–0.6740	0.7250	0.6894–0.7610	0.2310	0.2070–0.2540
RUSBoost	0.5000	0.4103–0.5910	0.4080	0.3394–0.4770	0.4060	0.3404–0.4720	0.6310	0.5912–0.6700	0.1640	0.1490–0.1790
Cost-NN	0.6360	0.5875–0.6850	0.8700	0.8148–0.9250	0.7980	0.7499–0.8460	0.8590	0.8269–0.8900	0.1020	0.0863–0.1170

Abbreviations: DC, dummy classifier; LR, logistic regression; RF, random forest; BRF, balanced random forest; XGBoost, extreme gradient boosting; RUSBoost, random undersampling adaboost; Cost-NN, cost-sensitive neural network.

**FIGURE 1:**
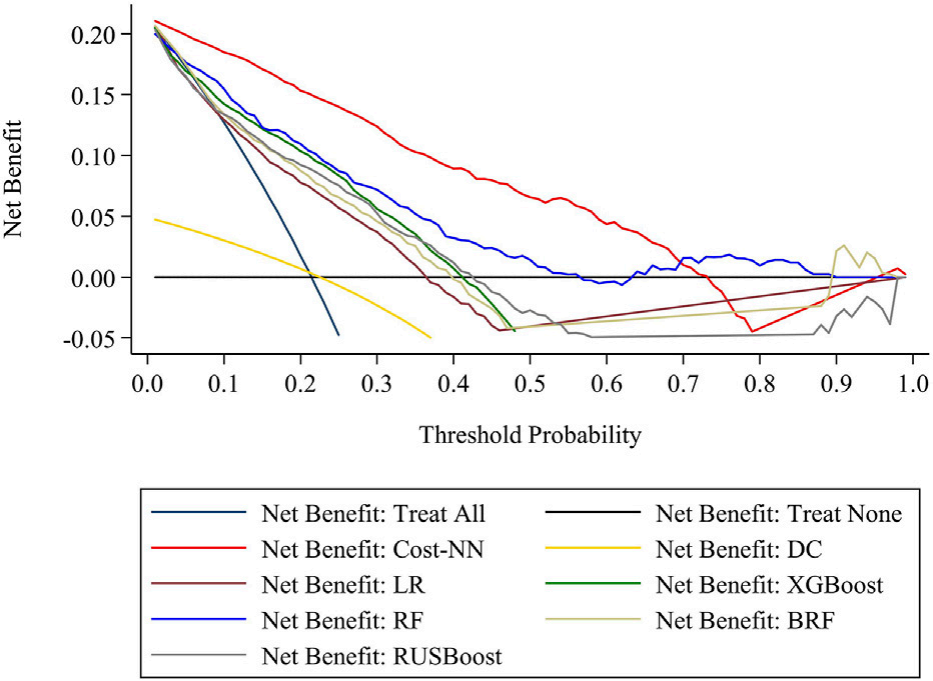
Decision curve analysis.

The model’s applicability in training practice was assessed using decision curve analysis, and the net benefit was corrected using cross-validation. As shown in Figure 1, it was evident that the prediction model of lower limb non-contact injury constructed by the Cost-NN algorithm has a higher area under the net benefit curve, which indicates that applying the risk prediction model of lower limb non-contact injury based on the Cost-NN algorithm to training practice will help to reduce the incidence of lower limb non-contact injury.

**FIGURE 2:**
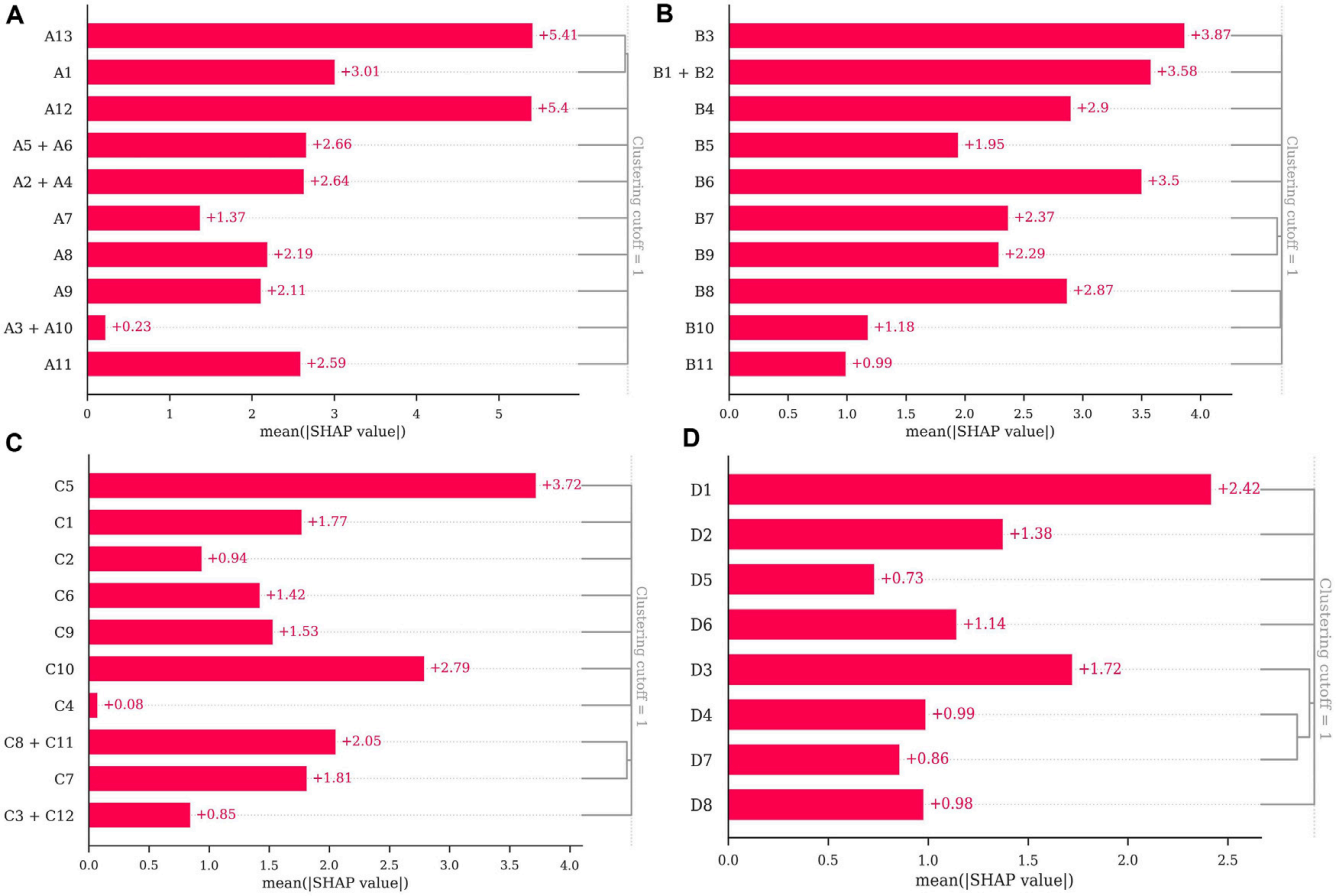
Feature importance of linear discriminant analysis: **(A)** agility attribute; **(B)** speed endurance attribute; **(C)** strength attribute; **(D)** speed attribute.

This study performed feature attribution analysis on the model to clarify each independent variable’s contribution to the model’s decision outcome. The hierarchical clustering analysis of feature importance in the linear discriminant analysis was performed using the SHAP. The results showed that urinary ketones (A13), urinary blood (A12), and the number of double under in 1 minute (A1) in the agility attribute test had greater importance **(** Figure 2A **)**. In the assessment of speed endurance attribute (Figure 2B), 1-minute HRR (B3) after 15 m × 13 shuttle run, 15 m × 13 shuttle run performance (B1), IRR after 15 m × 13 shuttle run (B2), and urinary protein (B6) had more significant importance. In the strength attribute test (Figure 2C), indicators such as the rate of creatine kinase change (C5) and urinary specific gravity (C10) had greater importance. The 3/4 basketball court sprint performance (D1), urinary protein (D3), and RPE (D2) in the speed attribute test had greater importance (Figure 2D).

**FIGURE 3:**
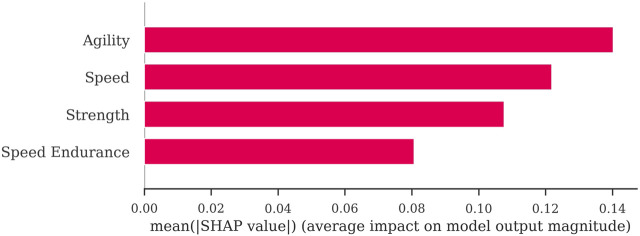
Feature importance of the injury risk prediction model based on cost-sensitive neural network.

Through the feature importance analysis of the injury risk prediction model based on Cost-NN, this study found that the agility attribute has the most significant feature importance, followed by the speed, strength, and speed endurance attributes (Figure 3). These results indicate that the agility attribute is an important feature affecting the model to predict the non-contact lower limb injury risk of youth female basketball players in Fujian Province.

## 4 Discussion

This study preliminary investigated the lower limb non-contact injury risk patterns of female youth basketball players in Fujian Province by retrospectively analyzing their sport-specific physical fitness monitoring database and injury registration database. The findings can provide theoretical references and analytical approaches for future research on injury risk patterns and developing non-contact injury prevention tools for lower limbs. There were two main findings from the study. Firstly, based on sport-specific physical fitness test data, the machine learning algorithm was used to develop the prediction model for lower limb non-contact injury risk. This model exhibited superior discrimination and calibration in identifying lower limb non-contact injury risk compared to the models typically utilized in research reports. Secondly, the model proposed in this study can provide practitioners with information on injury risk patterns. The feature attribution analysis identified the changes in agility and speed as important physical attributes influencing the lower limb non-contact injury risk of youth female basketball players in Fujian Province.

### 4.1 Lower limb non-contact injury risk prediction based on sport-specific physical fitness

Physical fitness monitoring is essential to a player’s training routine (McGuigan, 2017). Identifying players’ risk of sports injuries from physical fitness has become a topical issue in sports science research. However, the relationship between physical fitness and sports injuries is unclear due to heterogeneity between studies. Ruddy et al. (2019) point out that the misuse of statistical strategies is one reason for this problem. Ruddy et al. (2019) emphasized that regression has been widely used in sports science research due to its simplicity, reliability, and interpretability. However, regression has many assumptions (e.g., linearity and additivity), which has led to limitations in applying regression to injury risk prediction problems. Bittencourt et al. (2016) pointed out that, from the perspective of physics, the human body is an open system, which means that the occurrence of sports injury must result from the interaction and continuous development between the human body and the environment. Furthermore, the relationship between risk factors and injury outcomes is not always linear due to environmental influences, and there can be linear or non-linear interactions between injury risk factors. Thus, it is evident that regression is not the best approach to use for investigating injury risk prediction problems. To address this issue, some researchers have used machine learning algorithms to construct injury risk prediction models based on physical fitness tests and have achieved some valuable findings (López-Valenciano et al., 2017; Ruddy et al., 2018; Bryan et al., 2020; Jauhiainen et al., 2020; Rommers et al., 2020).

Therefore, based on sport-specific physical fitness, this study used machine learning to develop a model for predicting the risk of lower limb non-contact injuries in young female basketball players. Through comparative analysis, the model proposed in this study achieved a precision of approximately 63.6% in predicting the risk of lower limb non-contact injury, while the misdiagnosis rate was only 13%, which was significantly better than the baseline and models commonly reported in the literature such as logistic regression, balanced random forest, and XGBoost (López-Valenciano et al., 2017; Ruddy et al., 2018; Bryan et al., 2020; Jauhiainen et al., 2020; Rommers et al., 2020). It is important to note that this study used the occurrence of a lower-limb non-contact injury within the next 6 weeks as a dependent variable, which means that the injury outcome is precise. However, in the real world, the injury does not necessarily occur in the population at risk of injury. Meanwhile, different injury risk patterns may lead to similar injury outcomes (Huang et al., 2022). Therefore, it is suggested that the predictive performance of the model proposed in this study is acceptable. In addition, this study has further investigated the benefits of the model in practice by using the decision curve analysis. It can be observed that the lower limb non-contact injury risk prediction model proposed in this study has the largest area under the net benefit curve, which implies that the model can benefit players who have lower limb non-contact injury risk. These results indicate that the prediction model proposed in this study is a more effective and practical tool for lower limb non-contact injury risk assessment, which can provide coaches with the likelihood of an athlete’s lower limb non-contact injury in the following training period based on the stage physical fitness change patterns of athletes. Applying the model in training practice will help coaches pay attention to athletes’ physical fitness shortcomings and improve the periodized fitness training program on time, which is very important to reduce the sports injury rate.

### 4.2 Association between sport-specific physical fitness and lower limb non-contact injury risk

Which sport-specific physical fitness attributes were more strongly associated with lower limb non-contact injury risk patterns? This study investigated the relationship between specific physical fitness attributes and injury risk using feature attribution analysis of the model. It was found that agility and speed were important physical fitness attributes influencing the lower limb non-contact injury risk of young female basketball players in Fujian Province. The findings were similar to those found in male rugby players and teenage male football players (Quarrie et al., 2001; Caswell et al., 2016). Then, what were the patterns between agility attributes, speed attributes, and lower limb non-contact injury risk? This study found some patterns of change after analyzing the discriminant function of the LDA.

In the assessment of agility attribute, this study found that the likelihood of lower limb non-contact injury was significantly increased when players performed poorly on the hexagon agility test (more time to complete the hexagon agility test, higher IHR, and higher HRR) and had increased urinary protein, urobilinogen, urinary specific gravity, urinary blood, and urinary ketones after the agility attribute test. Among these, increased urinary blood and urinary ketones were important features that influenced the risk of lower limb non-contact injury. To our knowledge, this phenomenon can be explained from two perspectives. Firstly, from the perspective of energy metabolism in sports physiology, it reflects excessive intensity. According to the practical experience of training, after excluding female athletes who were menstruating, urinary protein and urinary blood indicators, which are positive after exercise, imply that the exercise intensity is excessive (especially for glycolytic energy-driven sports intensity). This finding may indicate that players have not yet adapted to the intensity of glycolytic energy-driven exercise, which makes them not yet develop the ability to change direction in line with competitive demands (Latzel et al., 2017). Secondly, from the sports biomechanics perspective, it could be that the player’s movements during the test were unreasonable, resulting in their movements being less efficient and increasing the energy metabolism losses. According to Cook et al. (2014), movement patterns that are poor not only lead to a decreased efficiency of the player’s movement but also increase the player’s injury risk during movement (Cook et al., 2014). Furthermore, there was an interesting finding in our study that the lower limb non-contact injury risk was increased when players performed well on their 1-min double under (Increase in the number of 1-min double under, decrease in IHR and HRR), which appears to be paradoxical. We speculate that this phenomenon could be linked to the inadequate maturation of the skeletal musculature in adolescent athletes. Although youth players’ physical performance and physiological adaptations have enhanced quickly after training, the delayed development of the skeletal-muscular system results in them having to carry additional mechanical loads.

In the assessment of speed attribute, this study noticed that players would have a significantly increased risk of lower limb non-contact injury when their 3/4 full-court sprint performance improved, similar to the findings by Bennett et al. (2022) in the Australian football program. Bennett et al. (2022) suggest that, on the one hand, players with a superior speed attribute may experience more acceleration and deceleration forces, which can increase stress on the skeletal-muscular system. On the other hand, players with a superior speed attribute may be involved in more training and competition, causing them to experience fatigue, which may impact the player’s athletic performance, increase recovery time and increase the likelihood of player injury (Chalmers et al., 2013; Ramos et al., 2019). However, the study by Bennett et al. could not provide physiological and biochemical response data after speed testing for evidence. Our findings revealed that there is a higher probability of lower limb non-contact injuries among players who experience a significant decrease in RPE, urinary protein, urinary specific gravity, and urinary ketone levels after the speed test, along with significant increases in urobilinogen and urinary blood levels. Among these, the decrease in RPE and urinary protein after speed attribute assessment were important features that influenced the risk of lower limb non-contact injury. This phenomenon is similar to the agility attribute results, meaning the physical function may not be well adapted to the increased mechanical loads associated with enhanced physical performance. These findings may provide physiological evidence to support the views of Bennett et al. (2022) and Chalmers et al. (2013) that players with a superior speed attribute may experience more acceleration and deceleration forces, leading to exhaustion due to increased loading on the musculoskeletal system, thereby increasing the likelihood of injury.

Although the link between sport-specific physical fitness and injury risk is controversial, our research suggests that there is an association between sport-specific physical fitness and injury risk that is not simply linear but a complex non-linear relationship, which may be modified by biological maturity. Some studies would support this speculation (Ramos et al., 2019). For example, Wilczyński et al. (2022) found a strong positive association between biological maturity, vertical jump, and distance long jump. A moderately positive association with dynamic balance and maturity offset in 72 healthy, youth male elite football players. In addition, le Gall et al. (2006) analyzed the relationship between biological maturity and injury incidence, severity, and distribution in 233 players. They found that injury incidence, severity, and distribution significantly differed between biological maturity subgroups. The results were similar to the findings by Monaco et al. (2018) on 164 football players. Steidl-Müller et al. (2020) found that changes in biological maturity and jump agility tests were important injury risk factors in 89 elite junior skiers. However, this study did not measure the biological maturity of youth female basketball players in Fujian Province to estimate its effect. Further research will be conducted to investigate the relationship between sport-specific physical fitness and injury risk in combination with biological maturity.

### 4.3 Limitations and perspectives

Despite these promising results, there are still limitations in this study. Firstly, the study sample size was limited. As this study aimed to investigate whether individual physical fitness change patterns impact the risk of lower limb non-contact injuries, only players affiliated with the same basketball team were analyzed in this study to avoid the effect of confounding factors such as age, gender, and training style. It is important to note that this team was affiliated with the Fujian Province Basketball and Volleyball Sports Management Centre, which means that the players were elite athletes in Fujian Province and can reflect the population characteristics of female youth basketball players in the region. However, the limited amount of data used in this study somewhat impacts the extrapolation value of the findings. Therefore, a multi-center prospective cohort study will be conducted to improve the extrapolation value of the findings in the future. Secondly, the measured indicators used in the study were limited. According to our understanding, the dataset used in this study was initially designed to evaluate sport-specific physical fitness developing trends in female youth basketball players rather than being applied to injury risk prediction and risk pattern research. Accordingly, the physical fitness testing protocols were mainly derived from field testing rather than laboratory conditions, which may impact the findings to some extent. It will be further investigated with laboratory tests (e.g., metabolomic and isometric muscle testing systems). Thirdly, the granularity of data for injury outcome variables remains large. Since different patterns of injury risk may lead to similar injury outcomes, there may be some limitations in using only binary variables as outcome variables. According to available research, parameters such as injury severity, the type of injury, and the actual time of injury occurrence can influence the injury risk pattern. Further studies, which take these variables into account, will need to be undertaken. In addition, it is worth noting that the problem of injury prediction is not the problem of simply classifying events as injury or non-injury, but rather a process of developing from non-injury to injury outcomes, so we suggest that the introduction of fuzzy mathematics will be able to promote the research in this area. Finally, the model proposed in this study still needs to be externally validated. As this study is a retrospective study based on historical data, there is still a lack of sufficient data to validate the external validity of the model. The data will be further collected, and the external validity of the model will be investigated.

## 5 Conclusion

This study preliminary investigated the relationship between sport-specific physical fitness change patterns and lower limb non-contact injury risk of female youth basketball players in Fujian Province using machine learning algorithms and field-based physical fitness tests, and proposed a lower limb non-contact injury risk prediction model. The model proposed in this study could effectively identify the lower limb non-contact injury risk of female youth basketball players in Fujian Province. Meanwhile, through model analysis, this study has identified change patterns in agility and speed attributes that impact the lower limb non-contact injury risk among youth female basketball players in Fujian Province, which were reflected not only in physical performance but also in physiological, biochemical, and subjective perceptual responses. These findings suggest that the player’s physical fitness change pattern can impact the lower limb non-contact injury risk. Although there are still many variables not taken into account, the findings and the data-driven model proposed in this study will provide valuable insights for fitness training program planning, fatigue management, and injury prevention in training practice.

## Data Availability

The original contributions presented in the study are included in the article/Supplementary Material, further inquiries can be directed to the corresponding author.
