# Supplementary material for: The impact of sport-specific physical fitness change patterns on lower limb non-contact injury risk in youth female basketball players: a pilot study based on field testing and machine learning
Source: Front Physiol. 2023 May 12;14:1182755. doi: 10.3389/fphys.2023.1182755 (PMC10213459; doi:10.3389/fphys.2023.1182755)
Supplement: Supplementary file 2 [file Table1.DOCX]

# Physical Performance Test Procedure

In accordance with the guidelines outlined in the Chinese youth basketball training syllabus, the Fujian Provincial Basketball and Volleyball Sports Management Center employed a battery of tests to evaluate the physical fitness attributes of the players. Specifically, the 15 m×13 shuttle run test was utilized to assess speed endurance, while the 3/4 basketball court sprint test was employed to evaluate speed. Agility was measured using the 1-minute double under and hexagonal agility test. The strength attribute of the players was assessed through a series of tests, including the 30-second 35kg squat, 30-second 20kg bench press, 30-second sit-up, and 30-second back extension tests. Details of the test follow as described:

# Speed Endurance

Speed endurance refers to the ability to sustain maximum velocity during multiple repetitions. Speed endurance is critical to basketball players. A player who can maintain the same level of speed in the second half of a game as they did in the first half demonstrates good speed endurance (Ratamess, 2011; Gamble, 2012; Turner, 2018). The 15m×13 shuttle run was used to measure the speed endurance attribute of the players. Players completed 13 runs between two basketball court sidelines for one test, with two tests conducted and a 5-minute rest period between each test. The best result was selected for analysis. Players were divided into three groups (5-6 people per group) for testing, monitored by 4 researchers using stopwatches. During the test, players were instructed to "cross the edge of the court" when turning. **The scorer reliability was observed during baseline for all participants in the hexagon jump (ICC = 0.979, 95%CI: 0.912–0.994).**

# Speed

The speed attribute refers to the ability to accelerate to maximum speed. Typically, a linear acceleration test measures a player's speed. Among these, the 3/4 basketball sprint is a standard test of speed attribute in basketball players since players will score in games through fast breaks (Gamble, 2012; Martin, 2016; Turner, 2018). Before the assessment commenced, the player was instructed to position themselves at the baseline of the basketball court. Upon hearing the command, the player will swiftly accelerate, dash across mid-court, and sprint toward the front-court free-throw line. Players were monitored by 4 researchers using stopwatches. **The scorer reliability was observed during baseline for all players in the hexagon jump (ICC = 0.986, 95%CI: 0.949–0.996).**

# Agility

Agility is the ability to change directions as quickly as possible. Therefore, the agility test assesses the player's ability to rapidly accelerate and decelerate quickly and change direction in a controlled manner using forward, backward, upward, and/or side shuffling movements (Ratamess, 2011). Therefore, this study used a 1-minute double under and hexagonal agility test to measure player agility attributes.

The 1-minute double under was used to assess agility in this study. This study measured the maximum number of movements in one minute to examine the achievement of double under. The players performed double under according to their styles (rhythm and timing). Before the experiment, they warmed up by performing exercises, including stretch and light jumping exercises. The same rope **(Asics, Kobe City, Japan)** was used for all attempts and adjusted to a suitable length for each player. The measurement was performed twice, and the higher value of the consecutive maximal number was used for analysis. Intervals between trials were set at 5 minutes, considering the influence of muscle fatigue.

The hexagonal agility test was commonly used for testing agility with restricted areas (Ratamess, 2011; Zhang et al., 2023). Players started in the center of a hexagon, facing the front line and with their feet together. On the start command, they jumped over the line and back to the center of the hexagon. They then jumped over the next side and back into the hexagon, repeating this process for three full rotations while facing forward. They had to keep the same direction and not land on the taped edges of the hexagon, or the attempt had to be restarted. The best score from two attempts was recorded. Players were monitored by 4 researchers using stopwatches. **The scorer reliability was observed during baseline for all players in the hexagon agility test (ICC = 0.989, 95%CI: 0.959–0.997).**

# Strength

Strength attribute refers to the ability of the human body's muscles to overcome external resistance when working. Strength attribute refers to the ability of the human body's muscles to overcome external resistance when working. This study used the 30-second 35kg squat, 30-second 20kg bench press, 30-second sit-up, and 30-second back extension test to evaluate the players' strength. There were two main reasons for this. Firstly, the musculoskeletal system of young players has yet to mature fully, and using standard 1RM muscle strength tests may impose additional stress on their musculoskeletal system, which can easily lead to injury (Martin, 2016). Secondly, muscle endurance is a major focus of strength training for young players, as developing muscle endurance can reduce the injury rate of players (Bergeron et al., 2015).

The 30-second squat test was used in this study to measure the players' lower body strength, where the barbell weight was set at 30kg. Before the assessment commences, players were mandated to assume a stance with their feet slightly wider than their hips' width and maintain an upright posture. The player was required to squat to the lowest position with the knee flexion angle not less than 90 degrees or until the leg muscles were parallel to the ground. The players were required to execute as many repetitions as possible within a 30-second.

The 30-second bench press test was used in this study to measure the players' upper body strength, where the barbell weight was set at 20kg. Before the assessment, the researchers instructed the players to lie on a flat bench with their legs on the ground, gripping a barbell with both hands at shoulder width and extending their elbows. In the bench press, the bar had to touch the chest on each repetition before returning to full arm extension, counting as one repetition. The players were required to execute as many repetitions as possible within a 30-second.

The 30-second sit-up test was used in this study to measure the players' abdominal strength. In the sit-up, Players lie flat on spongy pads with their feet on the mat and their hands behind their heads. Upon receiving the start command, they will sit up while ensuring their elbows touch the outside of both thighs. Subsequently, the players will recline, ensuring that their shoulders, back, and head are firmly in contact with the mat, completing one repetition. The players were tasked with executing as many repetitions as possible within a 30-second.

The 30-second back extensions test was used in this study to measure the player's lower back strength. In the back extensions, players lie face down with their hips supported on the bench and their ankles secured under the pads. Start with the player's torso down and bend 90 degrees at the waist. Upon receiving the start command, the player raises their body until their torso is parallel to the floor. Then return to the starting position and mark it as completed once. The players were tasked with executing as many repetitions as possible within a 30-second.

# References

Bergeron, M.F., Mountjoy, M., Armstrong, N., Chia, M., Cote, J., Emery, C.A., et al. (2015). International Olympic Committee consensus statement on youth athletic development. *British Journal of Sports Medicine.* 49, 843-851. doi: 10.1136/bjsports-2015-094962

Gamble, P. (2012). *Strength and Conditioning for Team Sports: Sport-Specific Physical Preparation for High Performance, Second Edition.* London: Taylor & Francis Group.

Martin, L. (2016). *Sports Performance Measurement and Analytics: The Science of Assessing Performance, Predicting Future Outcomes, Interpreting Statistical Models, and Market Value of Athletes.* Old Tappan: Pearson Education.

Ratamess, N. (2011). *ACSM's Foundations of Strength Training and Conditioning.* Indianapolis: Lippincott Williams & Wilkins.

Turner, A. (2018). *Routledge Handbook of Strength and Conditioning: Sport-Specific Programming for High Performance.* London: Taylor & Francis Group.

Zhang, M., Liang, X., Huang, W., Ding, S., Li, G., Zhang, W., et al. (2023). The effects of velocity-based versus percentage-based resistance training on athletic performances in sport-collegiate female basketball players. *Frontiers in Physiology.* 13. doi: 10.3389/fphys.2022.992655
